# Supplementary material for: Long non-coding RNA PSMB8-AS1 as a potential biomarker for postoperative recurrence in patients with Fuhrman grades 1–3 clear cell renal cell carcinoma
Source: PLoS One. 2026 Mar 11;21(3):e0343976. doi: 10.1371/journal.pone.0343976 (PMC12978458; doi:10.1371/journal.pone.0343976)
Supplement: S1 Table — (DOCX) [file pone.0343976.s001.docx]

# Table S1. Patient Characteristics

| Characteristics | Total | (%) |
| --- | --- | --- |
| Patients number | 192 |  |
| Age (years) |  |  |
| median | 67 (36-92) yrs |  |
| Gender |  |  |
| Male | 125 | 65.1 |
| Female | 67 | 34.9 |
| TNM stage |  |  |
| T1 | 137 | 71.35 |
| T2 | 7 | 3.65 |
| T3 | 47 | 24.48 |
| T4 | 1 | 0.5 |
| N0 | 190 | 98.96 |
| N1 | 2 | 1.04 |
| M0 | 186 | 96.88 |
| M1 | 6 | 3.12 |
| Fuhrman grade |  |  |
| G1 | 63 | 32.81 |
| G2 | 104 | 54.17 |
| G3 | 21 | 10.93 |
| G4 | 4 | 2.17 |
| Follow up duration |  |  |
| Median | 35.9 month |  |
| range | 1-2190 |  |
| Progression |  |  |
| No | 167 | 87 |
| Yes | 25 | 13 |
| Overall survival |  |  |
| survival | 177 | 92.2 |
| death | 15 | 7.8 |
