# Supplementary material for: Long non-coding RNA PSMB8-AS1 as a potential biomarker for postoperative recurrence in patients with Fuhrman grades 1–3 clear cell renal cell carcinoma
Source: PLoS One. 2026 Mar 11;21(3):e0343976. doi: 10.1371/journal.pone.0343976 (PMC12978458; doi:10.1371/journal.pone.0343976)
Supplement: S2 Table — (DOCX) [file pone.0343976.s002.docx]

# Table S2. Univariate and Multivariate Analysis to Predict Progression-Free Survival

## A. All patients; n=192

| Category | Comparison | Univariate HR (95% CI) | p-value | Multivariate HR (95% CI) | p-value |
| --- | --- | --- | --- | --- | --- |
| Gender | Female vs Male | 1.24 (0.89-1.72) | 0.2077 |  | 0.7016 |
| Age (median 65 yr) | Older vs Younger | 1.04 (0.46-2.34) | 0.9186 |  | 0.2117 |
| Stage - T | T34 vs T12 | 3.43 (1.54-7.67) | 0.0026 | 3.48 (1.46-8.31) | 0.0049 |
| Stage - N | N1+2 vs N0 | 20.61 (4.59-92.53) | <0.0001 | 5.40 (0.19-151.51) | 0.3217 |
| Stage - M | M1 vs M0 | 5.33 (1.55-18.30) | 0.0079 | 0.80 (0.16-10.48) | 0.8024 |
| Fuhrman grade | G4 vs G1+2+3 | 21.35 (5.81-74.49) | <0.0001 | 44.58 (4.26-466.98) | 0.0015 |
| NLR | 3< vs 3>= | 1.56 (0.65-3.78) | 0.3226 |  |  |
| lncRNA PSMB8 AS-1 | High vs Low (cut off: 17.4) | 3.34 (1.24-8.97) | 0.0163 | 4.29 (1.42-12.95) | 0.00098 |

## B. N0M0 Fuhrman G1-3; n=184

| Category | Comparison | Univariate HR (95% CI) | p-value | Multivariate HR (95% CI) | p-value |
| --- | --- | --- | --- | --- | --- |
| Gender | Female vs Male | 3.51 (1.33-9.23) | 0.0011 | 2.72 (1.01-7.31) | 0.0473 |
| Age (median 65 yr) | Older vs Younger | 0.78 (0.30-1.99) | 0.5992 |  |  |
| Stage - T | T34 vs T12 | 2.33 (0.93-5.80) | 0.0694 |  |  |
| Fuhrman grade | G3 vs G1+2 | 2.24 (0.71-6.79) | 0.1529 |  |  |
| NLR | 3< vs 3>= | 1.75 (0.66-4.64) | 0.259 |  |  |
| lncRNA PSMB8 AS-1 | High vs Low (cut off: 48.55) | 4.22 (1.66-10.74) | 0.0025 | 3.43 (1.32-8.92) | 0.0115 |

Abbreviations: RCC, renal cell carcinoma; HR, hazard ratio; CI, confidence interval.
