# Supplementary material for: Long non-coding RNA PSMB8-AS1 as a potential biomarker for postoperative recurrence in patients with Fuhrman grades 1–3 clear cell renal cell carcinoma
Source: PLoS One. 2026 Mar 11;21(3):e0343976. doi: 10.1371/journal.pone.0343976 (PMC12978458; doi:10.1371/journal.pone.0343976)
Supplement: S3 Table — (DOCX) [file pone.0343976.s003.docx]

# Table S3. Univariate and Multivariate Analysis to Predict Overall Survival

## A. All patients; n=192

| Category | Comparison | Univariate HR (95% CI) | p-value | Multivariate HR (95% CI) | p-value |
| --- | --- | --- | --- | --- | --- |
| Gender | Female vs Male | 0.75 (0.25-2.23) | 0.61 |  |  |
| Age (median 65 yr) | Older vs Younger | 2.20 (0.71-6.83) | 0.17 |  |  |
| Stage - T | T34 vs T12 | 4.21 (1.40-12.63) | 0.01 | 2.97 (0.10-1.11) | 0.0739 |
| Stage - N | N1+2 vs N0 | 211.42 (18.84-2371.51) | <0.0001 | 83731891 (0-) | 0.9992 |
| Stage - M | M1 vs M0 | 11.02 (2.96-41.01) | 0.0003 | 2.12 (0.25-18.06) | 0.4925 |
| Fuhrman grade | G4 vs G1+2+3 | 176.27 (14.79-2100.02) | <0.0001 | 6.485e-7 (0-) | 0.9994 |
| NLR | 3< vs 3>= | 0.92 (0.26-3.30) | 0.89 |  |  |
| lncRNA PSMB8 AS-1 | High vs Low (cut off: 17.4) | 2.40 (0.66-8.71) | 0.1843 |  |  |

## B. N0M0 Fuhrman G1-3; n=184

| Category | Comparison | Univariate HR (95% CI) | p-value | Multivariate HR (95% CI) | p-value |
| --- | --- | --- | --- | --- | --- |
| Gender | Female vs Male | 1.23 (0.33-4.59) | 0.7577 |  |  |
| Age (median 65 yr) | Older vs Younger | 1.23 (0.33-4.67) | 0.7575 |  |  |
| Stage - T | T34 vs T12 | 2.41 (0.69-8.43) | 0.1681 |  |  |
| Fuhrman grade | G3 vs G1+2 | 0.96 (0.12-7.69) | 0.9704 |  |  |
| NLR | 3< vs 3>= | 1.39 (0.36-5.39) | 0.6352 |  |  |
| lncRNA PSMB8 AS-1 | High vs Low (cut off: 48.55) | 1.36 (0.35-5.31) | 0.6567 |  |  |

Abbreviations: RCC, renal cell carcinoma; HR, hazard ratio; CI, confidence interval.
